# Supplementary material for: Causal Relationship between Aging and Anorexia Nervosa: A White-Matter-Microstructure-Mediated Mendelian Randomization Analysis
Source: Biomedicines. 2024 Aug 16;12(8):1874. doi: 10.3390/biomedicines12081874 (PMC11351342; doi:10.3390/biomedicines12081874)
Supplement: Supplementary file 1 [file biomedicines-12-01874-s001.zip › Supplementary Figure/Figure S9/Leaveoneout plot of MR analysis for ubm-b-1925 to AN.pdf]

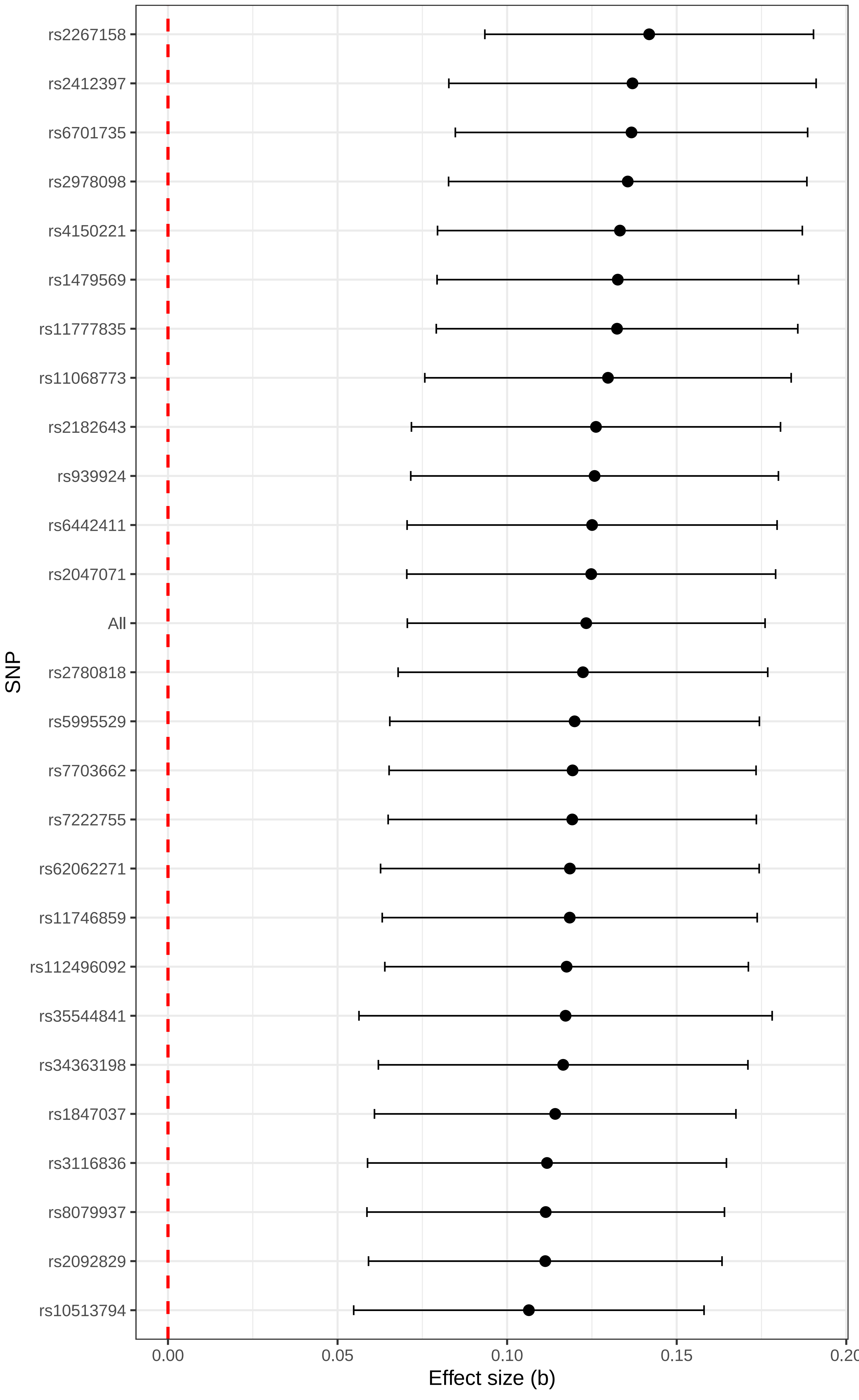
rs2267158
rs2412397
rs6701735
rs2978098
rs4150221
rs1479569
rs11777835
rs11068773
rs2182643
rs939924
rs6442411
rs2047071
All
rs2780818
rs5995529
rs7703662
rs7222755
rs62062271
rs11746859
rs112496092
rs35544841
rs34363198
rs1847037
rs3116836
rs8079937
rs2092829
rs10513794
SNP
0.00
0.05
0.10
0.15
0.20
Effect size (b)
